# Calaxin establishes basal body orientation and coordinates movement of monocilia in sea urchin embryos

**DOI:** 10.1038/s41598-017-10822-z

**Published:** 2017-09-07

**Authors:** Katsutoshi Mizuno, Kogiku Shiba, Junko Yaguchi, Daisuke Shibata, Shunsuke Yaguchi, Gérard Prulière, Janet Chenevert, Kazuo Inaba

**Affiliations:** 1 Shimoda Marine Research Center, University of Tsukuba, 5-10-1 Shimoda, Shizuoka, 415-0025 Japan; 2 Sorbonne Universités, UPMC Univ Paris 06 and CNRS, Laboratoire de Biologie du Développement de Villefranche-sur-mer, Observatoire Océanologique, 06230 Villefranche-sur-Mer, France; 3 Present Address: Center for Developmental Biology, RIKEN, 2-2-3 Minatojima-Minamimachi, Chuou-ku, Kobe, Hyogo, 650-0047 Japan

## Abstract

Through their coordinated alignment and beating, motile cilia generate directional fluid flow and organismal movement. While the mechanisms used by multiciliated epithelial tissues to achieve this coordination have been widely studied, much less is known about regulation of monociliated tissues such as those found in the vertebrate node and swimming planktonic larvae. Here, we show that a calcium sensor protein associated with outer arm dynein, calaxin, is a critical regulator for the coordinated movements of monocilia. Knockdown of *calaxin* gene in sea urchin embryos results in uncoordinated ciliary beating and defective directional movement of the embryos, but no apparent abnormality in axoneme ultrastructure. Examination of the beating cycle of individual calaxin-deficient cilia revealed a marked effect on the waveform and spatial range of ciliary bending. These findings indicate that calaxin-mediated regulation of ciliary beating is responsible for proper basal body orientation and ciliary alignment in fields of monociliated cells.

## Introduction

In vertebrates, two types of cilia are present in terms of the number of cilium per cell: monocilia and multicilia^1, 2^. Deficiencies in the formation and/or function of either type of cilium result in a group of disorders known as ciliopathies^1^ with multiple symptoms and devastating effects. Multicilia are present in epithelial tissues such as trachea, oviduct and brain where they are indispensable for producing a robust fluid flow for transport of several materials, particles and even cells. The direction of ciliary movement depends on the orientation of the basal body, which is primarily determined by the planar cell polarity (PCP) pathway during differentiation of epithelial tissues^3–5^. Coordination of ciliary movement as well as the orientations of basal bodies are highly responsive to the fluid-mediated hydrodynamic interactions between neighboring cilia^6, 7^. Initially, multiciliated cells are poorly polarized and their axonemes are randomly oriented. During tissue maturation, positive feedback due to the directional hydrodynamic flow created by early axonemal beating directs the progressive reorientation of cilia until all the axonemes of the cell beat in a unidirectional fashion^7^.

Monocilia are seen in the node, sensory organs, epithelia such as the renal epithelium, and spermatozoa (termed flagellum in the latter case). Most monocilia in human tissues are immotile primary or sensory cilia. In the node, there are two types of monocilia, immotile cilia on the crown cells and motile cilia on the pit cells. Nodal pit-cilia are tilted posteriorly and show rotary movements, resulting in directional fluid flow from right to left. Computational fluid dynamics and experimental observation demonstrate that the rotation of tilted cilia is the driving force for the leftward flow^8–10^. However, the roles of ciliary bend waveforms and how beating cooperation between neighboring monocilia is achieved are not well understood.

It has been demonstrated that Ca^2+^ is an important factor in the regulation of ciliary waveforms particularly in the case of spermatozoa, which are monociliated free cells. For example, sperm transiently change asymmetry of the flagellar waveform during chemotaxis to the egg in response to increase in the intracellular Ca^2+^ concentration^11–13^. A neuronal Ca^2+^ sensor family protein, calaxin, has been identified as the calcium sensor which regulates outer arm dynein during the propagation of asymmetric waveforms of sperm flagella in the ascidian *Ciona intestinalis* ^14, 15^. Because calaxin is an opisthokont-specific molecule and also present in ciliated cells other than sperm^14, 16^, we suspected that it may function in the Ca^2+^-dependent regulation of ciliary movements in epithelial tissues.

In this study, using sea urchin embryos, we investigated the function of calaxin in the regulation of monociliary movement. Although it is generally known that the outer arm dynein is essential for increasing beat frequency of cilia, morpholino knockdown experiment showed that calaxin is not directly related to the beat frequency. Intriguingly, the morphants did not swim due to disorganized beating direction. From a series of experiments, we found that calaxin is essential for establishing the orientation of ciliary basal structures.

## Results

### Coordination of ciliary movement is coupled with calaxin expression during embryonic development

**Figure 1:**
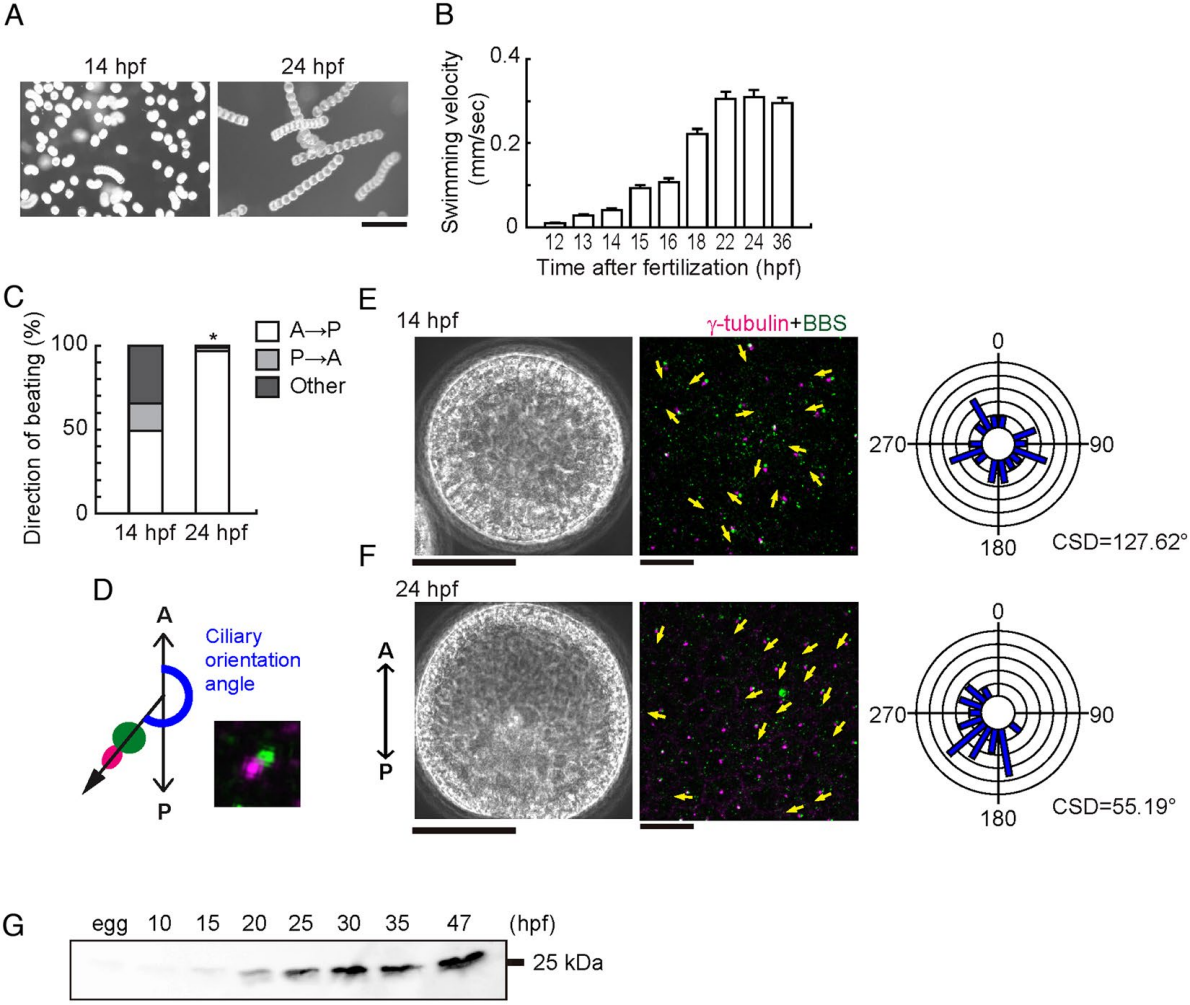
Ciliary beating direction and basal structure orientation are initially random and then become aligned. (**A**) Swimming trajectories of embryos. Ten images acquired at 0.3 second intervals are superimposed. hps, hours post fertilization. Scale, 0.5 mm. (**B**) Mean swimming velocities of embryos of different ages. N = 45–88 from 3–5 embryos. (**C**) Quantitative comparison of ciliary beating directions. A, anterior; P, posterior. N = 158 (14 h), 125 (24 h) from 8–9 embryos. *p < 0.001. (**D**) Schematic of angular analysis plotted in *E* and *F*. A vector drawn from the cilia transition zone (green) towards the centriole (magenta) gives the direction of ciliary basal structure (black) with respect to the anterior (A) - posterior (P) embryonic axis. A typical immunofluorescence image is shown. (**E**,**F**) Phase contrast (left), immunostaining of ciliary basal structures (middle) and circular histograms (right) in two representative embryos at 14 hpf (**E**) and 24 hpf (**F**). Yellow arrows indicate the direction in which ciliary basal structures are extended. Scale, 50 μm (left), 10 μm (middle). Circular histograms show the orientation of ciliary basal structures for 23 (14 hpf) and 22 (24 hpf) cilia. CSD, circular standard deviation. (**G**) Immunoblot of whole embryo proteins with anti-calaxin antibody.

Embryos and larvae of marine invertebrates bear motile monocilia on ectodermal cells and swim directionally due to their coordinated beating^17–19^. To investigate the regulation of coordinated movement of epithelial monocilia by calaxin, we took advantage of the sea urchin embryo model, which develop motile monocilia at the blastula stage for directional locomotion. First, we examined the motility of cilia, embryo swimming velocity and the orientation of the ciliary basal structure in the Japanese sea urchin *Hemicentrotus pulcherrimus* at different times after hatching. When cultured at 15 °C, *H. pulcherrimus* embryos start to hatch at ~12 hours post fertilization (hpf) and develop highly motile cilia on lateral cells. At that time, they lack a global forward movement and often swim rotationally (Fig. 1A; Supplementary Video S1). At ~14 hpf the embryos begin to swim linearly with a gradual increase in velocity to reach a maximum at ~24 hpf (Fig. 1A,B; Supplementary Video S2). We analyzed the beating of individual cilia using high speed camera and found that initially (14 hpf) the direction of ciliary beating is random with respect to the embryonic axis but by 24 hpf it becomes oriented in an anterior to posterior direction (Fig. 1C; Supplementary Videos S3 and S4).

In multiciliated cells, the beating direction of the cilia is determined by the orientation of basal bodies, more precisely of their accessory structures such as the rootlets and the basal feet^20^. A similar structural organization is known to be present in the monocilia of sea urchin embryos, giving an indication of basal body orientation and ciliary beating direction^21, 22^. To determine the orientation of ciliary basal structures in early embryos, we stained with anti-γ tubulin and anti-BBS1 (Bardet-Biedl syndrome 1) antibodies to localize centrioles and transition zone, respectively. In *H. pulcherrimus* embryos, BBS1 shows a distribution around the base of a cilium and γ-tubulin is localized to one edge of the BBS1 signal, thus allowing a clear visualization of basal body orientation (Fig. 1D). To quantify ciliary orientations relative to the embryonic axis, we measured the angle between a vector from the BBS1 signal to the γ-tubulin signal and the Anterior-Posterior (A-P) axis (Fig. 1D). Variations in ciliary orientations were expressed using mean value of circular standard deviation (CSD)^7^. We found that the ciliary basal structures are randomly oriented in 14 hpf embryos, but become aligned from the anterior towards the posterior direction with a slight tilt leftward in 24 hpf embryos (Fig. 1E,F; Supplementary Fig. S1). This alignment would be responsible for the forward locomotion with counterclockwise rotation (viewed from the posterior end) displayed by sea urchin larvae^17^.

To address the involvement of calaxin in ciliary cooperation, we next surveyed the expression of calaxin during embryonic development. We first isolated calaxin cDNA from *H. pulcherrimus* and prepared an antibody against recombinant calaxin (Supplementary Fig. S2). Immunoblots show that the calaxin protein starts to appear from 15 to 20 hpf and gradually increases during development (Fig. 1G), suggesting a possibility that the alignment of basal structures and the coordination of ciliary movement correlate with the increase of calaxin.

### Morpholino-knockdown of *calaxin* causes loss of ciliary coordination with no significant axonemal structure

**Figure 2:**
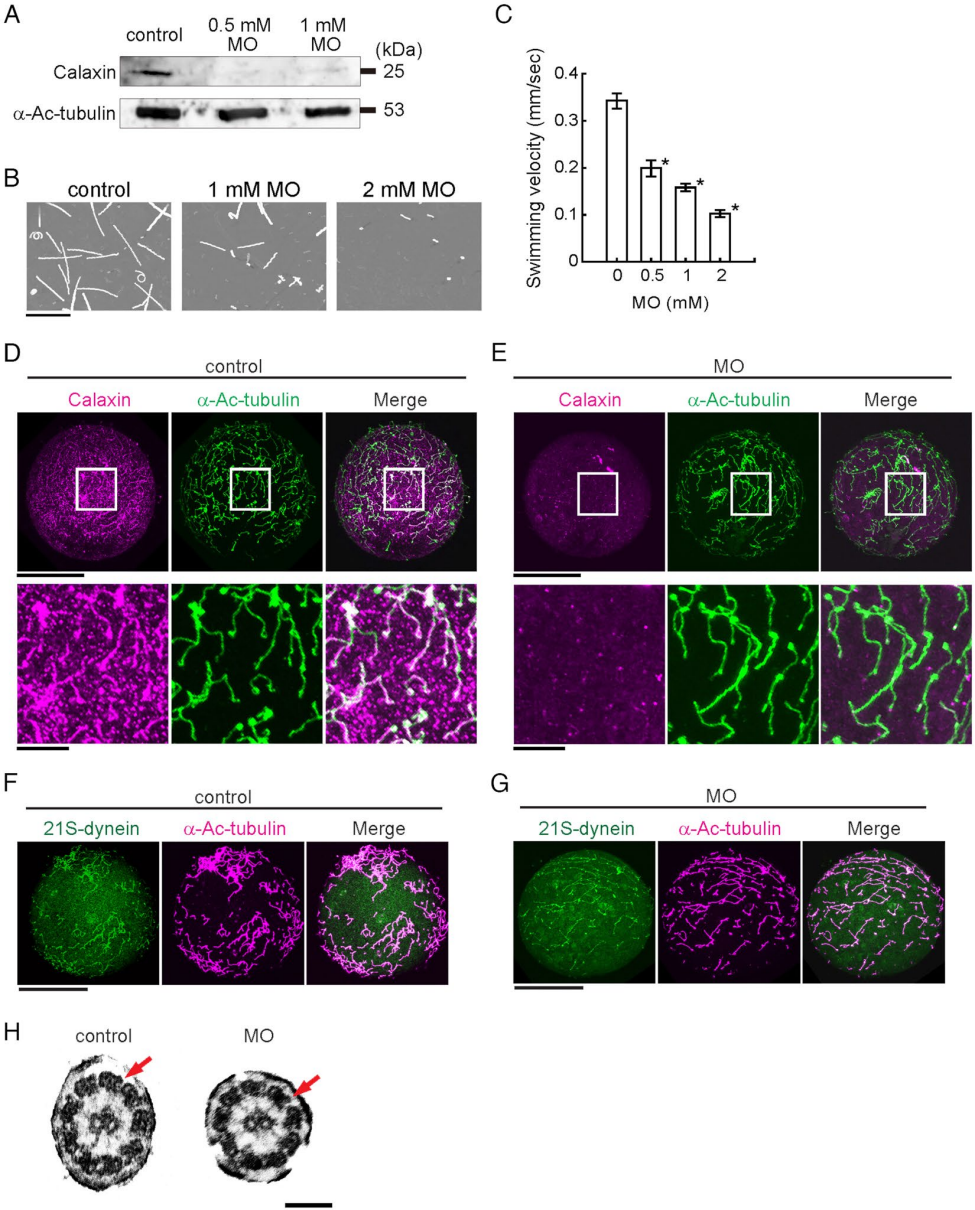
Knockdown of calaxin results in serious damage of embryonic swimming without changes in ciliary structures. (**A**) Immunoblots of control or MO-injected embryos (24 hpf) by anti-calaxin and anti-Ac-α-tubulin antibodies. (**B**) Swimming trajectories of sea urchin embryos at early gastrula stage; images acquired at 0.3 second intervals for 10 seconds are superimposed. Scale, 5 mm. (**C**) Comparison of mean swimming velocities of embryos injected with different concentrations of calaxin morpholino (MO). N = 61 (control), 38 (0.5 mM MO), 55 (1 mM MO), 28 (2 mM MO). *p < 0.001 vs control (0 mM). (**D**,**E**) Immunofluorescence comparison between control (**D**) and MO (1 mM)-injected (**E**) embryos. The upper rows, whole embryos; lower rows, enlarged images of squared regions. Scale, 50 μm (upper), 10 μm (lower). (**F**,**G**) Control embryo (**F**) and calaxin morphant (**G**) immunostained with antibodies against outer arm dynein, showing the presence of outer arm dyneins in morphant cilia. Scale, 50 μm. (**H**) Thin-section electron microscopy of cilia shows both outer (arrows) and inner arm dyneins in calaxin morphants. Scale, 100 nm.

To determine the function of calaxin during ciliary movement, we carried out knockdown experiments using morpholino (MO) injection, which drastically reduced calaxin levels (Fig. 2A). The morphants show impaired locomotion and move in a jerky fashion with decreased swimming velocity (Fig. 2B,C). Control embryos swim smoothly in a straight line at a velocity of ~0.34 μm/sec, whereas the morphants often spin on the bottom of the dish, resulting in a significantly lower swimming velocity (Supplementary Videos S5 and S6). However, the monocilia developed in morphants appeared comparable to those on control embryos on lateral cells (Fig. 2D,E) and apical tuft at the animal pole (Supplementary Fig. S3A). Immunofluorescent localization indicates that expression of calaxin is significantly decreased in morphant cilia (Fig. 2E). At the gastrula stage (27 hpf), the morphants develop normal animal and vegetal plates and mesenchymal cells, but gut formation fails (Supplementary Fig. S3B,C).

Because calaxin is associated with the outer arm dynein^14^, its knockdown may affect the formation or anchoring of outer arm dynein on the ciliary axoneme. To investigate this possibility, we immunolocalized dynein using an antibody raised against the whole outer arm dynein molecule^23^. This approach showed that in calaxin morphants, outer arm dynein was detectable along each cilium axoneme (Fig. 2F,G). Transmission electron microscopy confirmed that the distribution of outer arm dynein in the cilia of calaxin morphants appears normal (Fig. 2H).

### Knockdown of calaxin affects not ciliary beat frequency but ciliary bending and orientation of basal structures

**Figure 3:**
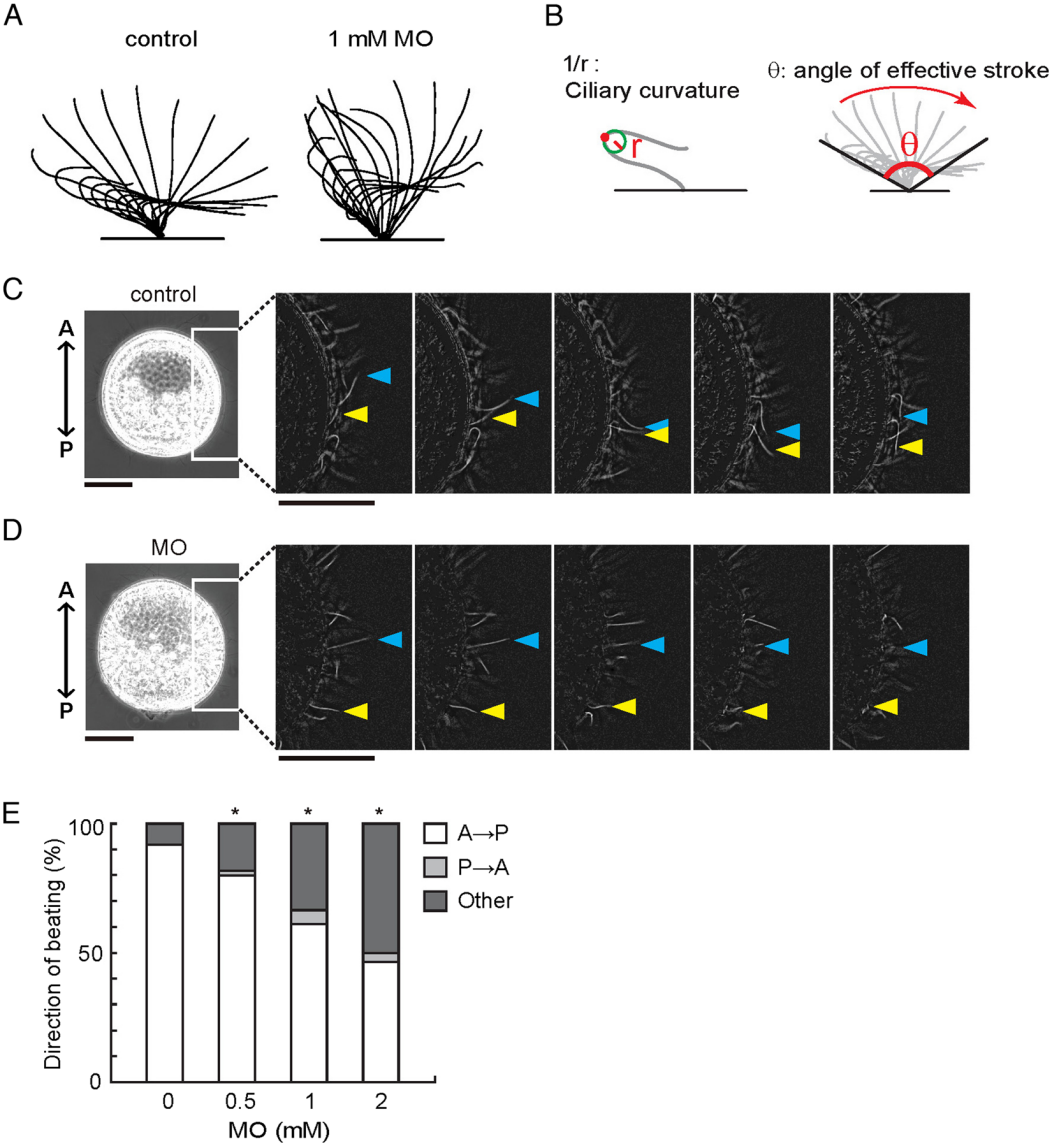
Lack of calaxin leads to disoriented ciliary movement with abnormal bend curvatures but normal beat frequency. (**A**) Typical waveforms during ciliary beating in control embryos and embryos injected with calaxin MO (1 mM). Motions of one cycle of beating are represented by the superimposition of images acquired at 5 msec intervals. (**B**) Definition of ciliary curvature and the angle of effective stroke. Both values were measured using video recordings of individual cilia and statistically analyzed as shown in Table 1. (**C**,**D**) Sequential images from high-speed videos (10 msec intervals) in a control embryo (**C**) and an embryo injected with calaxin MO (2 mM) (**D**). Arrowheads indicate the tip positions of individual cilia. Control cilia showed directional and coordinated beating but those from MO-injected embryo showed irregular ciliary beatings. Scale bar, 50 μm. (**E**) Quantitative comparison of ciliary beating directions, categorized as beating in anterior-posterior (A-P) direction, posterior-anterior direction (P-A) or other direction. N = 350 (control), 296 (0.5 mM MO), 424 (1 mM MO) and 212 (2 mM MO) from 16–29 embryos. *p < 0.001 vs control.

To further analyze the role of calaxin in ciliary motility, we next examined ciliary waveforms and their propagation. Outer arm dynein plays a role in increasing ciliary beat frequency^24^. As expected from the normal appearance of the outer arm dynein in the calaxin morphant, no significant change is observed in ciliary beat frequency (Table 1). Careful observation of the beating cycle of each cilium, however, revealed a marked effect of the absence of calaxin on the waveform and spatial range of ciliary bending (Fig. 3A): Cilia of calaxin morphants show a narrower range of effective stroke and less ciliary curvature in the recovery stroke (Fig. 3A,B; Table 1).

**Table 1:** Properties of ciliary beating in control embryos and in those injected with calaxin-MO.

	MO concentration [mM]			
	0	0.5	1	2
Beat frequency	9.78 ± 0.28	10.30 ± 0.22	10.10 ± 0.37	10.59 ± 0.33
(Hz)	(N = 53)	(N = 65)	(N = 60)	(N = 50)
Mean curvature	0.219 ± 0.004	0.209 ± 0.029	0.197 ± 0.031	0.189 ± 0.032
(µm^−1^)	(N = 40)	(N = 27)	(N = 43)*	(N = 23)**
Maximal curvature	0.426 ± 0.063	0.415 ± 0.074	0.402 ± 0.074	0.382 ± 0.011
(µm^−1^)	(N = 41)	(N = 25)	(N = 40)	(N = 23)
Angle of effective	145.8 ± 2.3	138.6 ± 3.5	131.0 ± 2.8	122.5 ± 3.6
stroke (degree)	(N = 39)	(N = 26)	(N = 41)**	(N = 24)**

Ciliary bending was analyzed for several successive cycles of beating. Values are mean ± standard error. N indicates the number of cilia. The values were compared with the Dunnett’s test. *p < 0.01, **p < 0.001.

Strikingly, the direction of ciliary beating is disorganized in the morphants. In control embryos, cilia on the lateral sides beat in a similar direction from anterior to posterior (A-P) (Fig. 3C; Supplementary Video S7). However, many of the cilia in the calaxin morphants beat in random directions with respect to the embryonic axis with no metachronal wave among cilia (Fig. 3D; Supplementary Video S8). The percentage of cilia beating in a direction parallel to the A-P axis decreased in a manner dependent on MO concentration (Fig. 3E), suggesting that calaxin-mediated regulation of ciliary motility coordinates the beating direction of cilia. The microinjection of mRNA encoding calaxin partially but significantly rescued both the swimming velocity of the embryos and ciliary beating direction (Supplementary Fig. S4A,B), suggesting that calaxin is required for coordination of ciliary beating. However, the gut formation was not recovered by mRNA injection in the morphants (Supplementary Fig. S4C).

**Figure 4:**
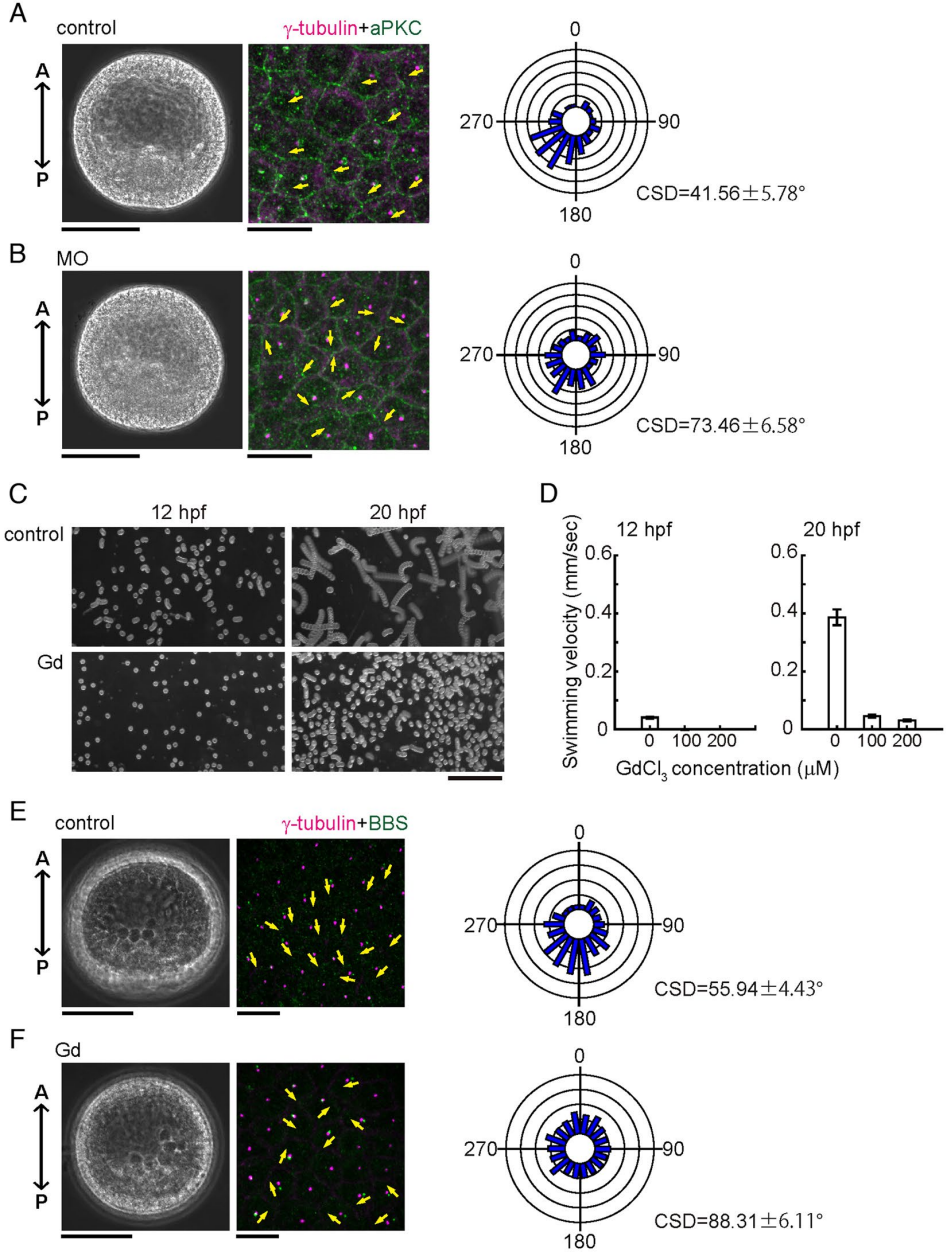
Calaxin morphants are deficient in the coordinated orientation of ciliary basal structures. (**A**,**B**) Phase contrast (left), immunostaining of ciliary basal structures (middle) and circular histograms (right) of control (**A**) and MO (2 mM)-injected (**B**) embryos at 24 hpf. Magenta, centrioles (γ-tubulin); green, transition zones (aPKC). Yellow arrows, direction of ciliary basal structures. Circular histograms show the orientation of ciliary basal structures. N = 89 (control) and 95 (2 mM MO), using 7–9 different embryos. Scale, 50 μm (left), 10 μm (middle). (**C**) Swimming trajectories of embryos treated with 100 μM GdCl_3_. 10 images acquired at 0.2 second intervals are superimposed. Scale, 1 mm. (**D**) Comparison of mean swimming velocities of control and Gd^3+^-treated embryos. N = 30–40 from 3 embryos. (**E**,**F**) Phase contrast (left), immunostaining of ciliary basal structures (middle) and circular histograms (right) of control (**E**) and Gd^3+^-treated (**F**) embryos at 20 hpf. Circular histogram, N = 502 (control) and 693 (100 μM Gd^3+^) using 24–25 different embryos. Scale, 50 μm (left), 10 μm (middle).

To determine if calaxin is also involved in the orientation of ciliary basal structures, we examined the distribution of two marker proteins, γ-tubulin and atypical protein kinase C (aPKC) which like BBS1 is localized at the base of cilia and forms a ring-like structure at the transition zone of cilia in the *Paracentrotus lividus* sea urchin embryo^22^. We found that in control embryos, basal bodies are mostly aligned in a parallel fashion (Fig. 4A). In contrast, their orientations become disordered in calaxin morphants (Fig. 4B). We quantified ciliary orientation as in Fig. 1D, by measuring the angle between the A-P axis and a vector from the center of the aPKC ring to the γ-tubulin signal. In control embryos, most ciliary basal structures are directed in a posterior direction, falling within the range of 180° − 270° (Fig. 4A), while cilia of calaxin morphants show randomized directions (Fig. 4B). CSD in calaxin morphants (73.46 ± 6.58, n = 7) is significantly larger than that in control embryos (41.56 ± 5.78, n = 9) (p < 0.001), demonstrating that the orientations of ciliary basal structures are disrupted by knockdown of calaxin.

### Ciliary beating is required to establish the orientation of basal structure

To examine whether the establishment of basal body orientation is coupled with normal ciliary motility, we investigated several chemicals for their effect on ciliary motility. Among those tested, we found that Gd^3+^, a stretch-activated ion channel blocker, strongly inhibits ciliary beating of 12 hpf embryos (Supplementary Fig. S5A; Supplementary Videos S9, S10) without causing significant morphological changes until mesenchymal blastula stage. Swimming of the blastula and ciliary beat frequency are significantly suppressed both at 12 and 20 hpf (Fig. 4C,D; Supplementary Fig. S5B). Immunostaining shows that the aligned orientation of basal bodies is largely disrupted in Gd^3+^-treated embryos at 20 hpf embryos (Fig. 4E,F); mean CSD in control and Gd^3+^-treated embryos were 55.94 ± 4.43 (number of embryos = 24) and 88.31 ± 6.11 (number of embryos = 25) (p < 0.001), respectively. These results indicate that proper ciliary beating at an early embryonic stage is prerequisite for establishment of ciliary orientation. Taken together, we conclude that the effect of calaxin on the establishment of basal body orientation is not by direct action on basal bodies but rather a consequence of ciliary beat regulation.

## Discussion

Our results clearly show that specific disruption of a Ca^2+^ sensor for outer arm dynein leads to disordered orientation of monocilia basal bodies without causing any apparent defects in the structure of outer arm dynein or axoneme. The cilia formed in calaxin morphants of sea urchin blastula have normal morphology and beat frequency but generate altered waveforms which show reduction in both the angle of effective stroke and the curvature of recovery stroke. Such changes in wave asymmetry coincide well with those observed during the chemotactic behavior of calaxin-suppressed sperm^15^. A similar reduction in ciliary waveform curvature is observed in flagella of *Chlamydomonas* mutants and those of human patients^25, 26^. All of these cilia with altered waveforms show slow movement or lower transport efficiency.

The most striking finding in this study is that calaxin morphants show deficiency in the alignment of basal body orientations. Comparison of parameters for ciliary beating between control embryos and calaxin morphants suggests that the establishment of ciliary orientation is driven not by elevation of beat frequency but by propagation of proper waveform during a beat cycle (Fig. 3A; Table 1). One beat cycle comprises an effective stroke and a recovery stroke. The former exerts strong viscous resistance, whereas the latter minimizes the resistance recovery stroke by a highly bent waveform. As a result, mechanical work by the former is approximately five times that of the latter^27^. Therefore, the reduced amplitude of the effective stroke angle and the disordered bending of the recovery stroke observed in calaxin morphant cilia are likely to induce diminished hydrodynamic thrust. Considering that the orientations of basal structure and beating of multicilia are thought to be determined by mechanical feedback of hydrodynamic force^5–7, 28^, calaxin-dependent regulation of ciliary waveform would be ultimately responsible for the alignment of the base structure in embryonic monocilia in sea urchins.

Because the function of calaxin depends on the intracellular Ca^2+^ concentration^14, 15^, the present study suggests that Ca^2+^ could be a signaling cue controlling coordinated ciliary motility in monocilia. The role of Ca^2+^ in the regulation of the ciliary waveform is well demonstrated in sperm and *Chlamydomonas* flagella^16^. In contrast, the potential roles of Ca^2+^ dynamics in the regulation of ciliary orientation and bending in nodal pit-cells have not been clarified. Gd^3+^ suppresses both coordinated ciliary motility and subsequent orientation of basal bodies in sea urchin embryo (Fig. 4C–F), implying a mechanistic similarity to the disruption of nodal flow by Gd^3+^ ^29^. Further studies on the roles of Ca^2+^-dependent regulation of ciliary waveform by calaxin should shed new light on the general regulatory mechanisms of motility and signaling in epithelial monocilia.

## Methods

### Animals and embryo culture

Adult sea urchins *Hemicentrotus pulcherrimus* were collected around Shimoda Marine Research Center (University of Tsukuba, Shizuoka, Japan), Marine and Coastal Research Center (Ochanomizu University, Chiba, Japan), and the Research Center for Marine Biology (Tohoku University, Asamushi, Japan). Spawning was induced by intrablastocoelar injection of 0.5 M KCl and the gametes were collected in microfiltered natural sea water (MFSW) or kept dry (sperm). After fertilization, embryos were cultured by standard methods in MFSW at 15 °C.

### Molecular cloning of Hp-calaxin

A TBLASTN search using the *Ciona intestinalis* calaxin sequence (Ci-calaxin) was carried out against the sea urchin *Strongylocentrotus purpuratus* Sp-base database (http://sugp.caltech.edu/SpBase/). SPU_007213, the sequence showing the highest degree of homology to *Ci-calaxin*, was used to design primers (5′-ATGGACAGAGTCCTGAGAGCA-3′ and 5′-CACTCTAACAGTAGCGGCTCAA-3′). A cDNA fragment was amplified by PCR from total RNA of 30 hours post-fertilization (hpf) *Hemicentrotus pulcherrimus* sea urchin embryos. The PCR product was subcloned into the pGEM-T Easy vector (Promega), and 5′-and 3′-RACEs were carried out to obtain the full-length cDNA sequence. The RACE primers 5′-CCTCGGTGGGTTGCTTTACCATCGATGTCT-3′ (5′-RACE) and 5′-ATGGTAAAGCAACCCACCGAGGAAG-3′ (3′-RACE) were designed based on the partial *Hp-calaxin* gene sequence.

### Microinjection of morpholino antisense oligonucleotide (MO) and mRNA

Morpholino antisense oligonucleotide Hp-calaxin MO was obtained from Gene Tools (Philomath, OR, USA). It was designed to cover the 5′-ATG region of the Hp-calaxin mRNA and had the following sequence: 5′-CAGCATTCTTATTACTTCCTTTCAT-3′. For injection, dejellied eggs were arrayed in rows on a 35-mm plastic dish coated with 1% protamine sulfate (Sigma). After insemination in FSW containing 3-amino-1,2,4-triazole (Sigma), microinjection was performed with a micromanipulator (Narishige) and an injector (Femtojet; Eppendorf)^30, 31^. Hp-calaxin MO was diluted at 0.5 mM to 2 mM final concentrations in 24% glycerol and injected at about 1% of the egg volume. Control embryos were injected with a corresponding volume of 24% glycerol. mRNA was synthesized from linearized plasmids using the mMessage mMachine kit (Thermo fisher Scientific) and injected at 20 ng/μl mRNA in 24% glycerol.

### Gd^3+^ treatment

For treatment with Gd^3+^, embryos were transferred to an artificial seawater (ASW; 423 mM NaCl, 9 mM KCl, 9.27 mM CaCl_2_, 22.94 mM MgCl_2_, 25.50 mM MgSO_4_, 2.14 mM NaHCO_3_, pH 8.2) containing several concentrations of GdCl_3_. The solution was centrifuged before use. Recording of embryonic swimming and ciliary motility were carried out at 12 hpf (just after treatment with Gd^3+^) and at 20 hpf.

### *In situ* hybridization

Whole mount *in situ* hybridization of embryos and larva was carried out as previously described^30^, except that the digoxygenin-labeled RNA probes for *endo16* and *SM50* were used.

### Preparation of antibodies and immunoblotting

A polyclonal antibody against Hp-calaxin was raised in mouse as previously performed^14^. PCR primers used for the amplification of Hp-calaxin open reading frame were 5′-GCGCGGATCCATGATGAAAGGAAGTAAT-3′ (sense) and 5′-GCGCGAATTCTCATCCGTCTCTTGCATT-3′ (antisense). The PCR product was subcloned into pET32a vector and transfected into *Escherichia coli* AD494. Protein expression was induced by 0.5 mM IPTG (isopropyl β-D-thiogalactoside). Thioredoxin–calaxin fusion protein was purified using Chelating Sepharose Fast Flow (GE healthcare). Thioredoxin-calaxin fusion protein at more than 95% purity was used for production of the antibody. Proteins of whole embryos at the desired stage were treated with SDS lysis buffer, separated by SDS-PAGE and transferred to a polyvinylidene difluoride membranes. Membranes were treated with 7.5% skim milk in PBST (PBS containing 0.1% Tween 20) to prevent non-specific protein binding. Blots were incubated with the anti-Hp-calaxin (1:5000) and anti-acetylated-α-tubulin (D20G3; Cell Signaling Technology, 1:5000) primary antibodies for 2 hours at room temperature. After washing with PBST four times, blots were incubated with HRP-conjugated secondary antibodies at 1:5000 for 1 hour at room temperature. After washing with PBST four times, blots were developed using the ECL-Plus enhanced chemiluminescence substrate kit (GE Healthcares). Signals were detected using the LAS-4000 mini imager (Fujifilm).

### Immunostaining and electron microscopy

For immunofluorescence detection, the anti-Hp-calaxin antibody raised in this study was used at 1:100. The following commercial antibodies were also used: anti-acetylated-α-tubulin (1:100, D20G3, Cell Signaling Technology), anti-γ-tubulin (1:500, GTU 88; Sigma-Aldrich), anti-aPKC (1:200, SC216, rabbit polyclonal C-20; Santa Cruz Biotechnology), anti-BBS1 (1:200, ab111847, Abcam). Immunostaining was performed based on a previously described method with minor modifications^22^. Embryos were fixed in −20 °C cold methanol containing 10 mM EGTA (ethylene glycol tetraacetic acid), pH 7.5 for 10 min. For staining with anti-BBS1 antibody, embryos were pre-fixed in 36.2 mM EGTA and 1% formaldehyde before fixation by cold methanol. Fixed samples were washed three times with PBS-0.05% Triton X-100 for 15 min then blocked with 3% BSA (bovine serum albumin, 015-15103; Wako) for 2 hours at room temperature. Incubation with primary and secondary antibodies was carried out for 2 h at room temperature. The primary antibodies were detected with secondary antibodies conjugated with Alexa-488 and Alexa-546 (Invitrogen). The specimens were observed by confocal microscopy (Fluoview FV10i, Olympus). Electron microscopy was performed as described previously^14^.

### Analysis of embryo swimming behavior and ciliary beating

To observe swimming behavior, sea urchin embryos were kept in seawater on 35-mm plastic dishes coated with BSA and observed at 25 °C using a binocular microscope (MZ12.5; Leica) equipped with a digital camera (HDR-CX700; Sony). Swimming velocities were calculated with BohBoh software (Bohboh soft). To observe movements of individual cilia, embryos were immobilized between a glass slide and a coverslip separated by 58-µm-thick double stick tape (3 M Scotch) and observed using a phase contrast microscope (BX51; Olympus) equipped with a high-speed camera (200 frames per seconds, HAS-220; DITECT) as previously described^31^. For analysis, embryos with a lateral orientation (Anterior-Posterior axis parallel to the coverslip) were selected. Concerning 14 hpf embryos, the side covered with cilia was regarded as posterior side and its opposite side as anterior. Because the cilia of the animal region (apical tuft) are long and immotile, only movements of cilia from lateral regions of embryos were analyzed. The direction of cilium movement was determined by following the focal point of the cilium tip over time and was classified as A-P (anterior side to posterior side), P-A (posterior side to anterior side), or others (ciliary tip becoming out of focus). Several cycles of ciliary movements were analyzed using Bohboh software, which traced ciliary waveform and calculated beat frequency, maximum curvature, and angle of effective stroke.

### Determination of the angle between ciliary basal structure and embryonic A-P axis

The angle was measured on images of gastrulae double-labelled with anti-γ-tubulin antibodies and either anti-aPKC or anti-BBS1. For each cell, a line between the center of the BBS1 (aPKC) signal and the γ-tubulin signal was drawn and the angle between this vector and the embryonic axis from posterior to anterior was calculated using Bohboh software.

### Statistical analysis

Data were compared using the Dunnett’s test for multiple comparison such as comparison of swimming velocity, and the t-test for comparison of two independent groups. The percentages of AP directional beating were compared with Fisher’s exact test (Fig. 1C and Fig. 3E) and Tukey’s multiple comparison test (Supplementary Fig. S4). For Fig. 3E, the result was adjusted with multiple comparison correction using Holm’s method. For circular data analysis, Oriana software (Kovach Computing Services) was utilized. Circular standard deviation was calculated as described previously^7^.

## Electronic supplementary material

Supplementary information

Supplementary Video S1

Supplementary Video S2

Supplementary Video S3

Supplementary Video S4

Supplementary Video S5

Supplementary Video S6

Supplementary Video S7

Supplementary Video S8

Supplementary Video S9

Supplementary Video S10
