# Supplementary material for: Calaxin establishes basal body orientation and coordinates movement of monocilia in sea urchin embryos
Source: Sci Rep. 2017 Sep 7;7:10751. doi: 10.1038/s41598-017-10822-z (PMC5589754; doi:10.1038/s41598-017-10822-z)
Supplement: Supplementary file 1 — Supplementary information [file 41598_2017_10822_MOESM1_ESM.pdf]

Supplementary Materials:

Calaxin establishes basal body orientation and coordinates movement of monocilia in sea urchin embryos

Katsutoshi Mizuno, Kogiku Shiba, Junko Yaguchi, Daisuke Shibata,

Shunsuke Yaguchi, Gérard Prulière, Janet Chenevert and Kazuo Inaba

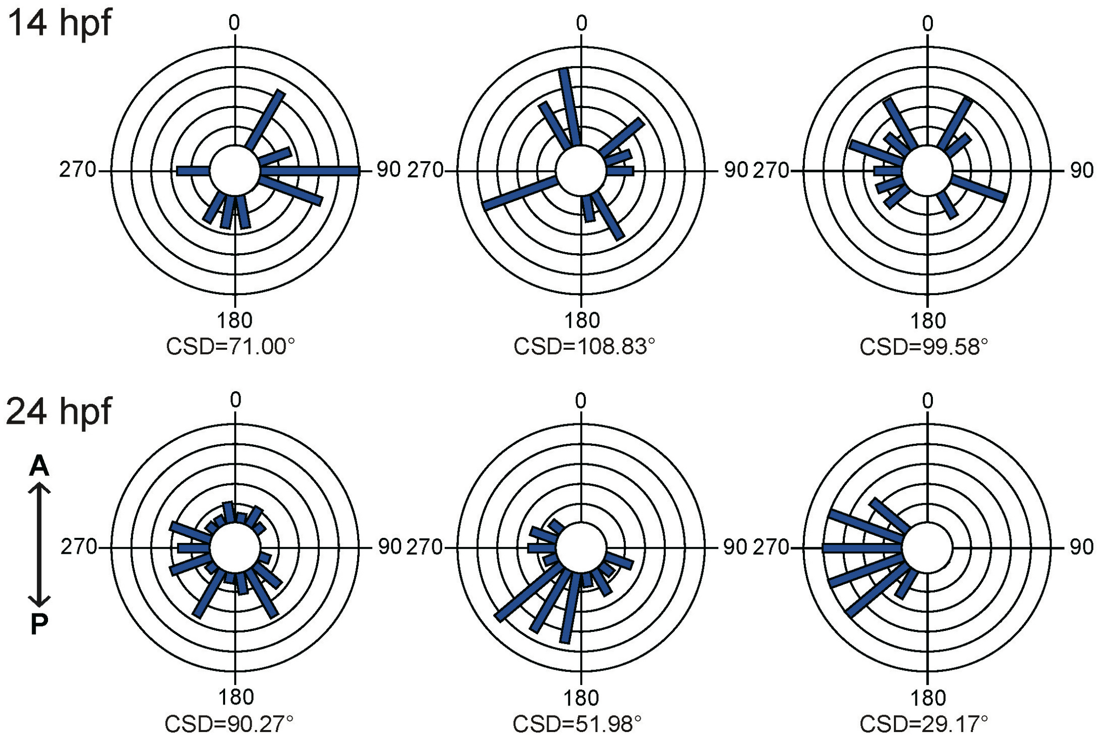

Supplementary Figure S1. Circular histograms showing the orientation of ciliary basal structures. Data were obtained from 3 embryos at 14 hpf and 24 hpf after fertilization. The number of cilia analyzed = 12-15 (14 hpf) and 25-37 (24 hpf) for each embryo. CSD, circular standard deviation.

A

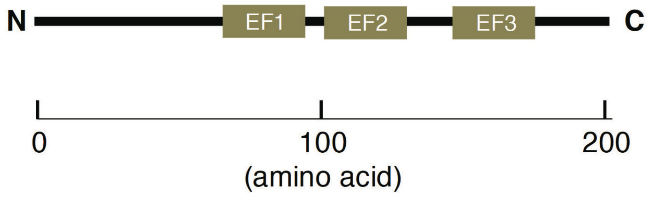

B

```
tunicate       -MSKKNQKLAEELYKTSCQKHFTKTEVESLIICYKNLLEGLK-------MDRNLFRDILH
human          MNRKKLQKLTDTLTKN--CKHFNKFEVNCLIKLFYDLVGGVERQGLVVGLDRNAFRNILH
sea_urchin     ----MMKGSNKNAVHRLKDCHFSKEEQESLVKMFKELTKENTGSIREDKLDRSQFREVLH
                     :   .   :     **.* * :.*:  : :*            :**. **::**

tunicate       QKFNMTEDLLMDRVFRAFDKDSDSYISLTEWVEGLSVFLRGTLDEKMEYTFTVFDLNGDG
human          VTFGMTDDMIMDRVFRGFDKDNDGCVNVLEWIHGLSLFLRGSLEEKMKYCFEVFDLNGDG
sea_urchin     SIFKMTDDVIMDRVLRAFDKDNDACINLYEWLTGLSVFLKGSLEEKIQFCFKVFDLNSDG
                 * **:*::****:*.****.*. :.: **: ***:**:*:*:**::: * *****.**

tunicate       YISREEMFQMLKTCLVKQPTEEDPDEGIKDLVEIALKKMDHDHDSRLSKKDFKDAVLIEP
human          FISKEEMFHMLKNSLLKQPSEEDPDEGIKDLVEITLKKMDHDHDGKLSFADYELAVREET
sea_urchin     FISREEMFHLLKTSMVKQPTEEDPEEGIKDLVEIIIKKMDLDHDGKLSFKDFCDSVNVSR
               :**:****::**..::***:****:********* :**** ***.:**  *:  :*  .

tunicate       LLLEAFGKCLPDEKSSEIFEYHVLGVKQCRG--
human          LLLEAFGPCLPDPKSQMEFEAQVFKDPNEFNDM
sea_urchin     YSWSVSGHAYRSRRLVTISWGNARDG-------
                  .. * .  . :       :.
```

C

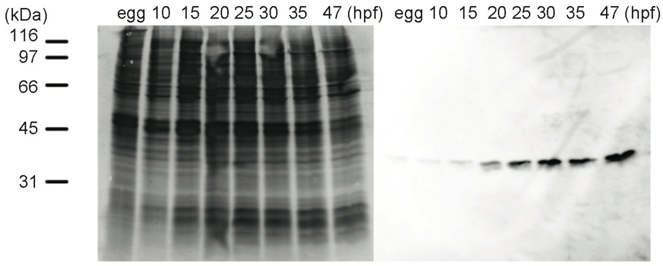

Supplementary Figure S2. Domain structure and expression during early embryonic development of sea urchin calaxin. (*A*) Location of EF-hand motifs in sea urchin calaxin. A calaxin cDNA from the sea urchin *Hemicentrotus pulcherrimus* (accession number, AB986239) contains 1,056 base pairs with an open reading frame encoding 202 amino acids. EF-hand motifs found by SMART search are located at positions 66-94, 102-130, and 147-175. (*B*) Multiple alignment of Hp-calaxin with calaxins from the tunicate *Ciona intestinalis* (AB079059) and human (NP_078869). Asterisks, colons, or dots indicate identical residues in all sequences, conserved substitutions, or semi-conserved substitutions, respectively. Shaded areas show the positions of EF-hand motifs. (*C*) Immunoblots with anti-calaxin antibody on whole embryo proteins at hours post fertilization (hpf) indicated. Egg, unfertilized egg. Left, protein staining; right, immunoblot.

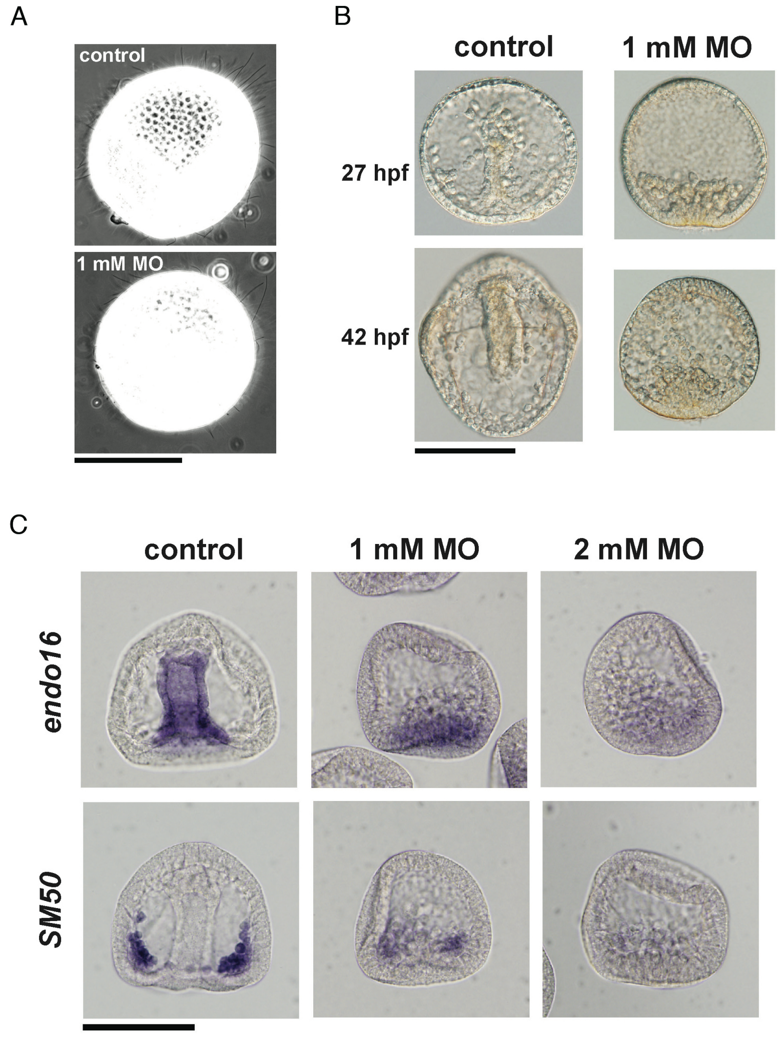

Supplementary Figure S3. Loss of gut formation in calaxin morphant. (*A*) Phase contrast images of control and MO-injected embryos at 24 hpf embryos, showing the formations of apical ciliary tuft and lateral cilia are normal in the morphant. (*B*) Differential interference contrast images of control and MO-injected embryos at 27- and 42 hpf. The formation of gut is not observed in the calaxin morphants, but a mass of cells is internalized and retained on the posterior base of the blastocoel at both 27- and 42 hpf. (*C*) Expression of an endoderm marker gene (*endo16*) and primary mesenchyme marker gene (*SM50*) at 24 hpf, detected by whole mount *in situ* hybridization. *endo16* is expressed in gut in normal embryos, whereas it is expressed at the cellular mass on the posterior base of the blastocoel in the morphant (1 mM MO). *SM50* is expressed in primary mesenchyme in normal embryos; A weaker signal is detected in the mesenchymal cells of the morphant (1 mM MO). For both genes, expression become less detectable in embryos injected with 2 mM MO. Scale bar, 100 μm.

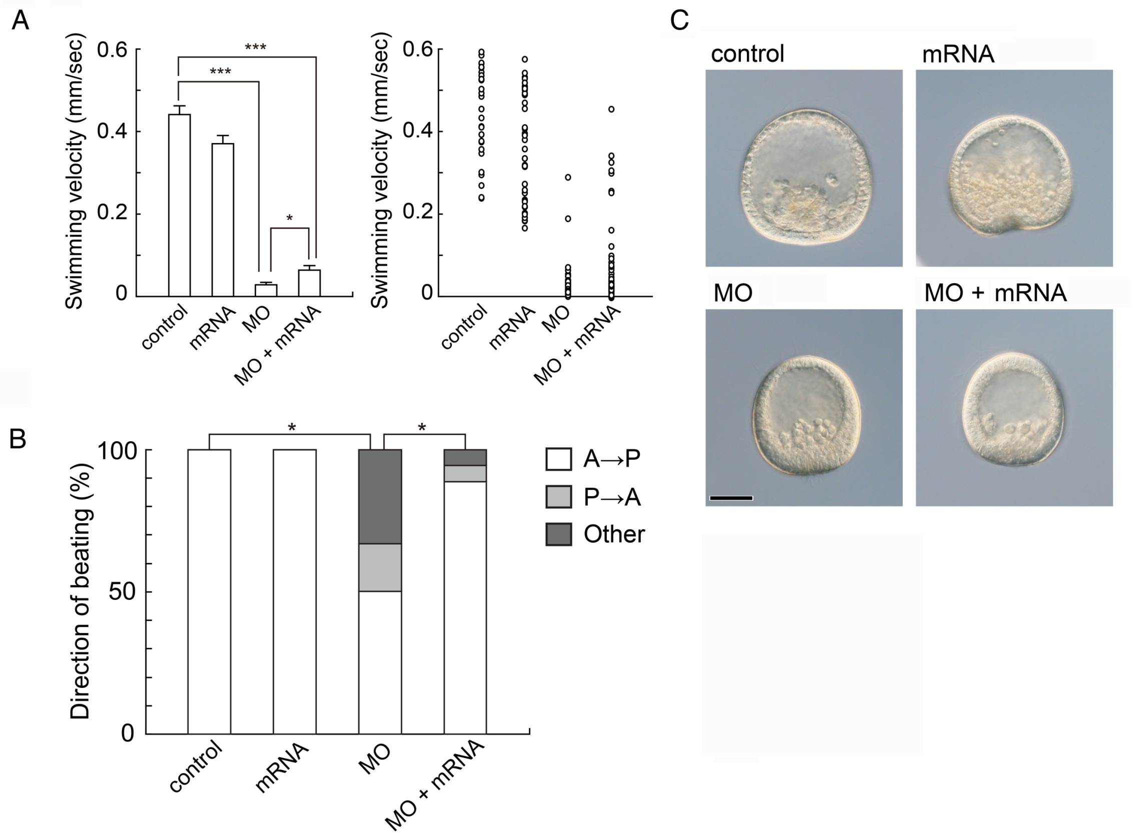

Supplementary Figure S4. Rescue of swimming velocity and ciliary beating orientation of embryos by injection of *calaxin*-mRNA. (*A*) Comparison of mean swimming velocities of embryos (left) and plots of swimming velocities from individual embryos (right). Mean ± s.e.m. N=27 (control), 42 (20 ng/μl mRNA), 57 (2 mM MO), 72 (2 mM MO and 20 ng/μl mRNA). *: $p < 0.05$ or ***: $p < 0.001$. (*B*) Quantitative comparison of ciliary beating directions, categorized into anterior-posterior (A-P) direction, posterior-anterior direction (P-A) or other direction. N=160 (control), 152 (20 ng/μl mRNA), 159 (2 mM MO), 177 (2 mM MO and 20 ng/μl mRNA) from 8-10 embryos. The percentages of AP directional beating were compared by Tukey's multiple comparison test. *: $p < 0.001$. (*C*) Differential interference contrast images of control, mRNA-injected, MO-injected, and MO+mRNA-injected embryos at 24 hpf. Bar, 100 μm.

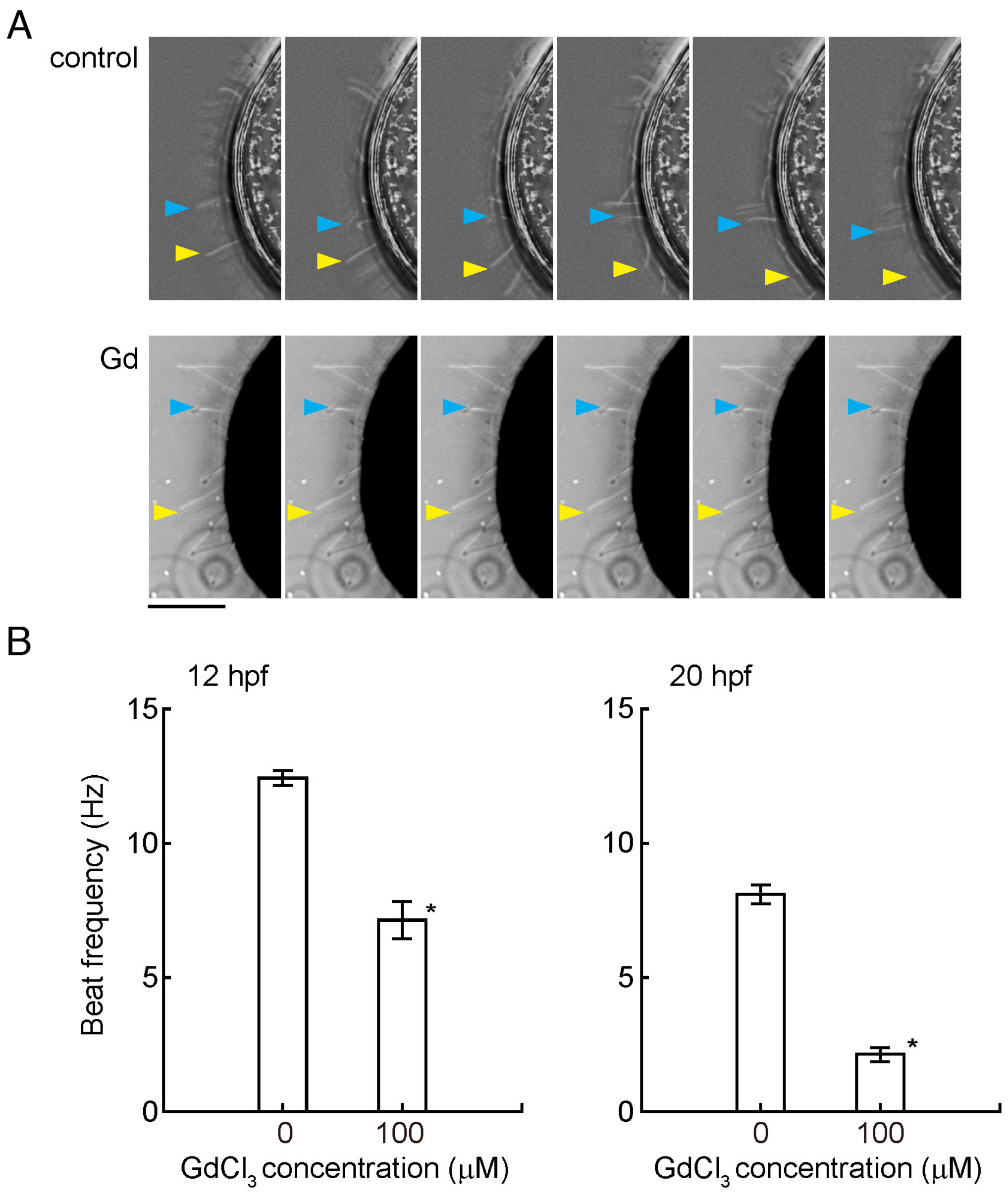

Supplementary Figure S5. Suppression of ciliary beating in sea urchin embryos by Gd^{3+}. The embryos were treated with 100 μM $GdCl_3$ at 12 hpf. (*A*) Ciliary beating recorded in a control and a Gd^{3+}-treated embryo 5 min after treatment. Sequential images from high-speed videos (10 msec intervals) are shown. Blue and yellow arrowheads indicate the tip positions of two individual cilia for each condition. Scale bar, 20 μm. (*B*) Comparison of mean ciliary beat frequency. Gd^{3+}-treated embryos showed significant decrease in beat frequencies at 12 and 20 hpf. N=11-18 from 3 embryos. Statistical significance was determined by t-test. * $p < 0.001$ vs control (0 μM).

Supplementary Video S1. Swimming behavior of sea urchin embryos just after hatching (14 hpf, dark-field image). The movie plays at real time.

Supplementary Video S2. Swimming behavior of sea urchin embryos 10 hours after hatching (24 hpf, dark-field image). The movie plays at real time.

Supplementary Video S3. Beating pattern of cilia on sea urchin embryo just after hatching (14 hpf, phase contrast image). The anterior-posterior axis is not clear at this developmental stage. The movie plays at 0.15X speed.

Supplementary Video S4. Beating pattern of cilia on sea urchin embryo 10 hours after hatching (24 hpf, phase contrast image). Top, anterior end; bottom, posterior end. The movie plays at 0.15X speed.

Supplementary Video S5. Swimming behavior of normal sea urchin embryo (24 hpf). The movie plays at real time.

Supplementary Video S6. Swimming behavior of calaxin morphants (2 mM MO injected, 24 hpf). The movie plays at real time.

Supplementary Video S7. Ciliary movement in normal sea urchin embryos (24 hpf). Ciliary movement was observed by high-speed camera. Phase contrast image was inversed and the background was subtracted to increase the contrast of cilia. The movie plays at 0.15X speed.

Supplementary Video S8. Ciliary movement in calaxin morphant (24 hpf). Ciliary movement was observed by high-speed camera. Phase contrast image was inversed and the background was subtracted to increase the contrast of cilia. The movie plays at 0.15X speed.

Supplementary Video S9. Ciliary movement of 12 hpf embryo. Ciliary movement was observed by high-speed camera under a phase-contrast microscope. The movie plays at 0.15X speed.

Supplementary Video S10. Ciliary movement of 12 hpf embryo in the presence of 100 μM $GdCl_3$. Ciliary movement was observed by high-speed camera under a phase-contrast microscope. The movie plays at 0.15X speed.
